# Charge transport variation from Bloch–Grüneisen to Mott variable range hopping and transport change due to hydrogenation in Palladium thin films

**DOI:** 10.1038/s41598-021-01787-1

**Published:** 2021-11-16

**Authors:** Adithya Jayakumar, Viney Dixit, Sarath Jose, Vinayak B. Kamble, D. Jaiswal-Nagar

**Affiliations:** School of Physics, IISER Thiruvananthapuram, Vithura, Kerala 695551 India

**Keywords:** Materials science, Nanoscience and technology, Physics

## Abstract

We report a systematic investigation of the differences in charge transport mechanism in ultra-thin nano-island like films of palladium with thickness varying between 5 nm and 3 nm. The thicker films were found to be metallic in a large temperature range with a dominant Bloch–Grüneisen mechanism of charge transport arising due to electron-acoustic phonon scattering. These films were also found to exhibit an additional electron–magnon scattering. At temperatures below 20 K, the two films displayed a metal-insulator transition which was explained using Al’tshuler’s model of increased scattering in disordered conductors. The thinner films were insulating and were found to exhibit Mott’s variable range hopping mechanism of charge transport. The thinnest film showed a linear decrease of resistance with an increase in temperature in the entire temperature range. The island-like thin films were found to display very different response to hydrogenation at room temperature where the metallic films were found to display a decrease of resistance while the insulating films were found to have an increase of resistance. The decrease of resistance was ascribed to a hydrogen induced lattice expansion in the thin films that were at the percolation threshold while the resistance increase to an increase in work function of the films due to an increased adsorption of the hydrogen atoms at the surface sites of palladium.

## Introduction

Charge transport in low dimensional materials has received increasing attention due to the fact that quantum confinement affects play a prominent role at length scales that are comparable to or less than the de-Broglie length of the material^1–6^. At length scales less than nanometers, charge transport is likely to be quantum mechanical in nature^1–3,6^. However, for a large size range that are of the order of few nanometers, quantum transport may not be an issue. For metals at few nanometer scale, understanding charge transport is quite important since metallic interconnects in electronic devices are likely to have nanometre size in future devices. In this regard, a lot of investigation is going on in trying to understand the charge transport behaviour of metals other than Copper in order to overcome the problems associated with Copper as an interconnect metal^7–9^. Many studies have come up that try to understand the effect of specific electron scattering mechanism, the effect of surface scattering, grain boundary scattering etc. on the charge transport behaviour of metals^10–12^.

Since a lot of devices get fabricated in thin metal film forms, it is very important to investigate the changes to the charge transport mechanism of the films when the thickness of the films have been reduced to nanometre sizes. At such thicknesses, the electron scattering has additional contributions, arising from surfaces, defects, roughness, orientation of grains, grain boundary density etc.^8,13,15–18,66^. These parameters effect the residual resistivity of a metal and a knowledge of the variation of the residual resistivity with the thickness of the film may help tune the residual resistivity according to the need of a given device. As the thickness of a metal film is reduced such that the film transforms from a continuous thin film to that of an island-like configuration, it is expected that an associated change also happens to the charge transport mechanism from metallic to that of an insulator due to quantum confinement affects arising from an interplay of opposing effects of energy scales and length scales^4,13,14^.

Bulk palladium (Pd) is a metal which has been investigated extensively for its catalytic activity and selectivity towards hydrogen (H$_2$) gas^19–21^. At room temperature, metal Pd exists in a face centred cubic (fcc) lattice that forms palladium hydride (PdH$_x$) when exposed to hydrogen gas. Due to the catalytic activity of the Pd metal, the adsorbed H$_2$ gas molecules on the Pd surface dissociate into *H* atoms and permeate into the octahedral sites of the lattice increasing the lattice constant of fcc Pd due to the formation of fcc PdH$_x$^22–25^. For x < 0.015, a solid solution called the $\alpha$-phase is formed where the lattice expansion happens by about 0.15$\%$. Above x = 0.015, the solid solution transforms to a beta phase (PdH$_{\beta }$) where an increase in lattice constant by 3.4$\%$ takes place^22–25^. However when the Pd metal is in a thin film form, the catalytic activity of Pd’s surface is expected to change due to incorporation of additional defect centres present in the thin film^13,26,27^. In fact, films that are in the form of nano-islands may be at the percolative limit for some thickness and hydrogenation in such films may result in very different behaviour of resistance than for the films that are for from the percolative limit. Even though the gas sensing properties of hydrogen using Pd both in the bulk as well as nano form has been investigated quite intensively, the effect of hydrogen on the charge transport mechanism in Pd has not been studied so much.

For bulk metals, the established theory of charge transport is that of Bloch–Grüneisen which considers the electron scattering as arising due to electron–phonon interaction^28^. A linear decrease of resistance drops to that of a T$^5$ dependence below a characteristic Debye temperature $\Theta$ due to a loss of phase space resulting in a reduction of the electron scattering^29^. It is, however, not very well understood how the Bloch–Grüneisen mechanism would change when the metal is stabilised in a thin film form. Al’tshuler et al.^2,3^ have predicted that when the metal is in the thin film form, additional scattering centres like defects, surfaces etc. would result in dephasing of the electronic wave-function resulting in an increase in resistance at low temperatures. In this work, we have investigated the details of charge transport in nano-island like ultra-thin films of Pd whose thickness was varied from 5 nm to 3 nm. For the films of thickness 5 nm and 4.75 nm, the charge transport was found to be metallic for temperatures till $\sim$ 20 K where Bloch–Grüneisen mode of charge transport was found to be true. At lower temperatures, an increase in resistivity was observed that was explained by Al’tshuler’s model of defect induced scattering. For the films of lower thicknesses of 4.5 nm and 4 nm, the resistance showed an insulating behaviour where the charge transport was found to be of Mott’s variable range hopping kind. The lowest 3 nm thickness film was found to have a linear temperature variation with a negative slope in the entire temperature range. Room temperature resistance behaviour of the films of varying thickness was found to vary drastically upon hydrogen loading and unloading depending on the thickness of a given film. For thicker films of thickness 5 nm to 4.25 nm, the resistance was found to decrease upon hydrogen loading, possibly, due to a hydrogen induced lattice expansion of these films that have inter-island separation very close to a percolation threshold. A percolation model that was introduced in our earlier work^13^ was used to understand the details of the time-constants associated with the opening up of new percolative paths. On the other hand, for thinner films of 4 nm and 3nm thickness, the resistance was found to increase due to an increase in the work-function of the films on account of increased adsorption of the H atoms at the Pd surface sites.

## Experimental details

It is known that in evaporative deposition, a solid is heated to its evaporation temperature such that the evaporated material deposits over all solid angles in the vacuum chamber. The film morphology is governed by the condensation kinetics and thermodynamics of the seed that first forms on the substrate from the vapour phase. Typically, metallic films formed using thermal or e-beam assisted evaporation result in dense films with larger grain size^30,32^. In contrast, sputtering is a fast particle bombardment process where ions strike a solid surface and knock out the surface atoms through momentum transfer. This results in a plasma which consists of sputtering gas ions, electrons and knocked out energetic atomic species or atom clusters with smaller grain size^30,32^. The efficiency of the process is dependent on the energy of the sputtering ions, surface energy of the target material as well as mass ratio of the target atoms and sputtering ions, resulting in granular films. Hence, the granularity of the films can be controlled by the sputtering rate, substrate temperature and vapor pressure of the target material. The surface roughness of such granular films were found to be a very important parameter in controlling the functionality of thin metallic films, wherein, sputtered films performed better than those grown by vapour deposition^30–32^. Therefore, to get better control over particle size and inter-particle separation, we chose to use sputtering technique for making ultra-thin palladium films.

**Figure 1:**
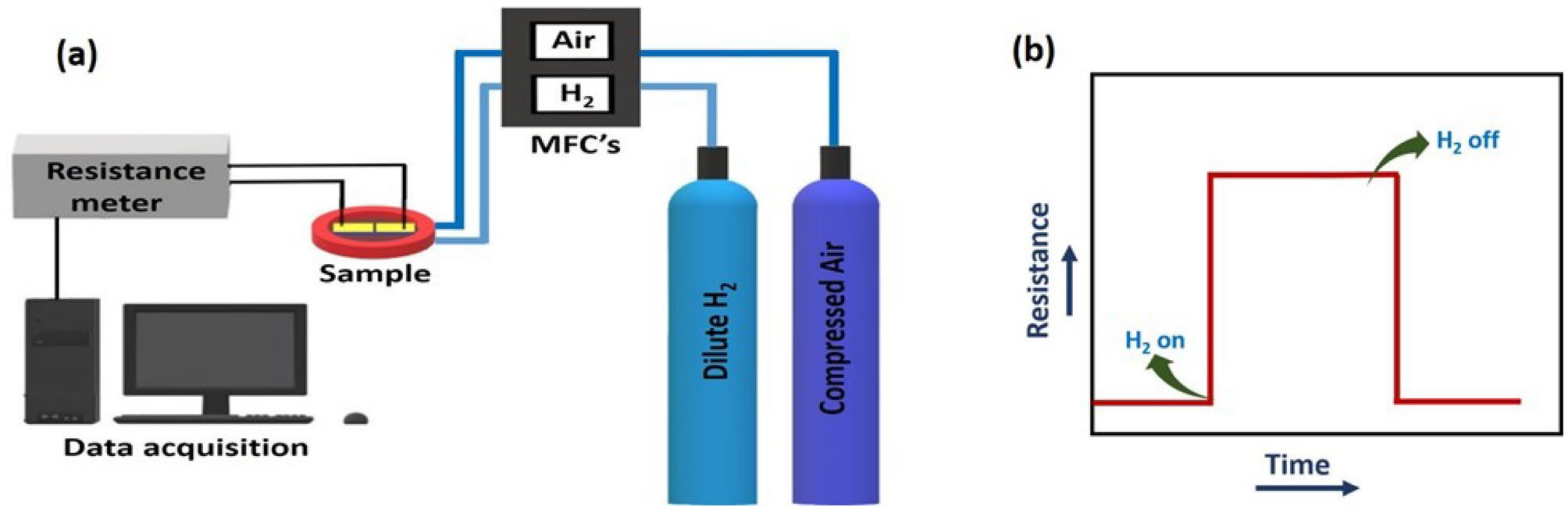
(**a**) Schematic of the set-up used for measuring the change in resistance on hydrogenation and dehydrogenation. Sample to be measured, measuring gas cylinders, mass flow controllers, measurment unit as well as data acquisition system have been shown. (**b**) Schematic of change in resistance upon hydrogenation and dehydrogenation has been shown as a function of time.

It is very well known that deposition conditions have a profound effect on the properties of the grown films^33–35^. By depositing TiN$_x$ films using reactive sputtering in a nitrogen environment, Ponon et al. found the conductivity of the films to improve by increasing the nitrogen content^33^. Similarly, theoretical calculations done on studying the effect of deposition conditions on the charge transport of an organic light emiting diode (OLED) showed that lower substrate temperature and higher initial velocity of inserting molecules resulted in better ordered morphology of the OLED resulting in higher electron mobility than hole mobility^34,35^. Hence, in order to study the charge transport of palladium films, it is very important to control the parameters of deposition very well. For this study, palladium was deposited in ultra-thin film form on prefabricated substrates by using radio frequency sputtering under varying control parameters. The sputtering power, gas flow rate etc. were controlled in order to get ultra-thin palladium nanostructured films as shown in Fig. 3a. The working pressure of the deposition chamber was set at 3 $\times$ 10$^{-3}$ mbar. The sputtering power was fixed to 5 W and the time of deposition was varied to obtain thin films of varying thickness. To calibrate the thickness of the palladium films to deposition time, a step was made on a thick film that was deposited for 60 min. The thickness was, then, measured using a profilometer. The defined values of thickness are “nominal” since thickness would be poorly defined in a quasi-continuous films comprising nanoislands either widely separated or sitting close to a percolation transition. Prefabricated substrates for the deposition of the films were prepared by cutting a glass slide into rectangular pieces of dimension 10 mm $\times$ 2.5 mm. The glass substrate was, then, dipped in a solution of chloro(dimethyl)-octylsilane for 1 h to ensure the formation of a thin self-assembled monolayer (SAM) shown as green colour in Fig. 3a. The substrate was then shadow masked with copper wire of diameter 100 micron and coated with a gold layer of thickness 50 nm over a wetting layer of chromium of thickness 10 nm by thermal evaporation. The masking provided a 80 $\upmu$m wide channel onto which palladium was deposited. The gold layer on either side of the narrow channel was used as contact pads as shown in Fig. 3a.

Temperature (T) dependent resistance (R) measurements were performed on a commercial Nanomagnetics Hall effect system’s closed cycle refrigerator. We confirmed the linearity of the I–V curves at 300 K and 5 K in the ± 100 mV range before measuring R–T. The effect of hydrogenation on the resistance of the palladium films was studied using an in-house built set-up^13,36,37^ shown schematically in Fig. 1a. The sample to be measured is shown in orange and has an incoming H$_2$ gas from a 1$\%$ dilute hydrogen cylinder (shown in blue). The base-line resistance of the sample is measured in air coming from an air cylinder (shown in purple). The flow of H$_2$ as well as air towards the measurement chamber was regulated using Alicat Scientific’s mass flow controllers (MFC’s) to achieve desired dilution of the H$_2$ gas with synthetic air. The change of resistance of the palladium films upon hydrogenation was measured using Keithley’s electrometer (Model No. 6517B) cum data acquisition system. Fig. 1b is a schematic plot of the change in resistance on hydrogenation and dehydrogenation measured as a function of time.

## Results and discussion

### Morphology, growth and scattering in ultra-thin films

**Figure 2:**
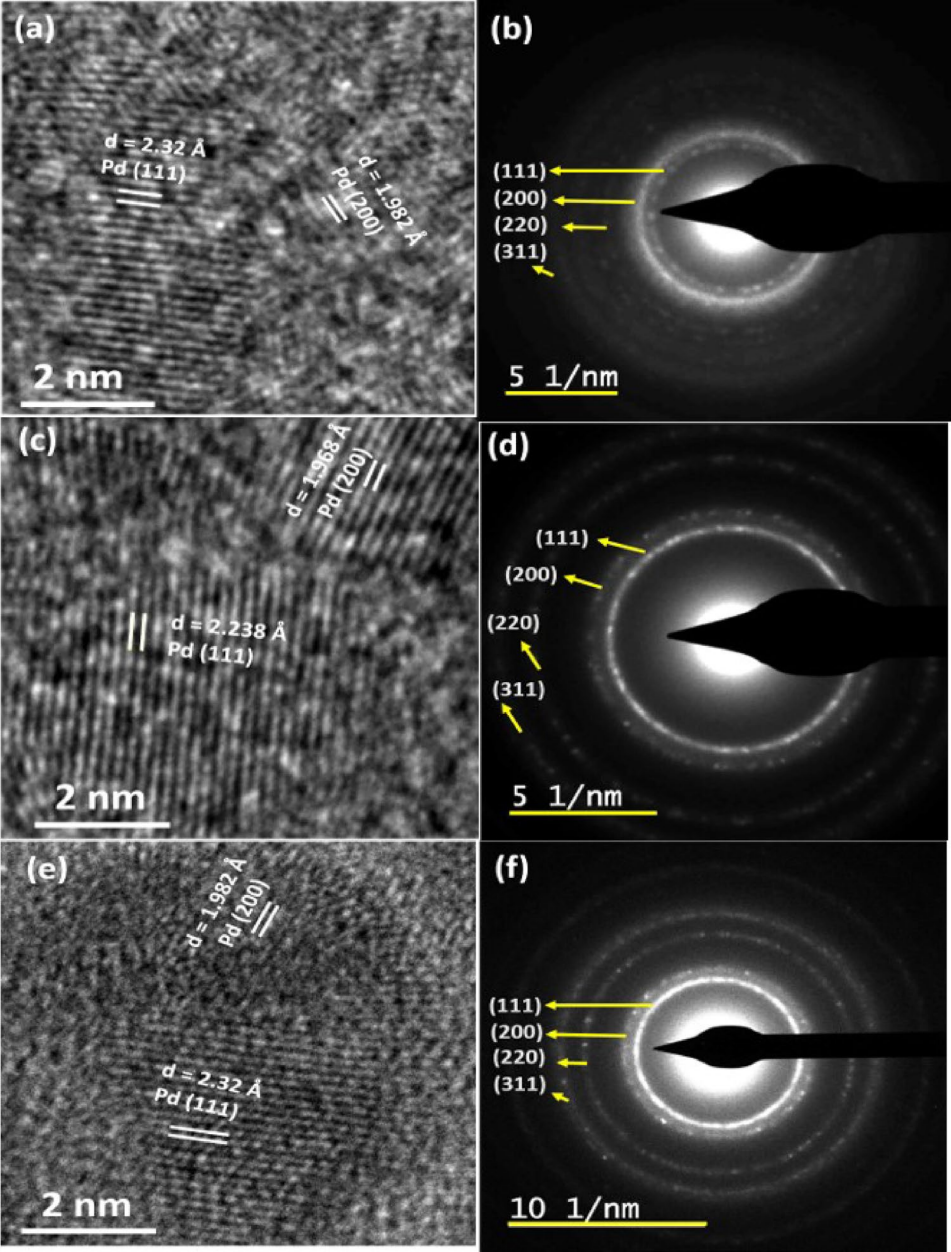
HRTEM micrographs of (**a**) 4.25 nm, (**c**) 4.5 nm and (**e**) 5 nm thin films measured at a 2 nm resolution. Lattice planes have been marked by white lines and distances corresponding to the Pd lattice shown. (**b**), (**d**) and (**f**) represent SAED images corresponding to HRTEM images of (**a**), (**c**) and (**e**) respectively. Lattice planes corresponding to the rings have been marked by arrows and shown.

In order to characterise the grown ultra-thin films, we performed both transmission electron microscopy as well as atomic force microscopy on them. Figure 2a–f show few representative HRTEM images obtained on films that were grown using a deposition time of (a) 4 min. 15 s, (c) 4 min. 45 s and (e) 5 min. The HRTEM images for the remaining two films are similar. From the HRTEM images, one can clearly see the formation of a uniform Pd lattice, wherein, (200) and (111) planes of Pd can be observed that have been marked at interplanar spacings of 1.98 $\AA$ and 2.32 $\AA$, respectively^13,38,39^. Figure 2b,d,f show the select area electron diffraction (SAED) images corresponding to the HRTEM

**Figure 3:**
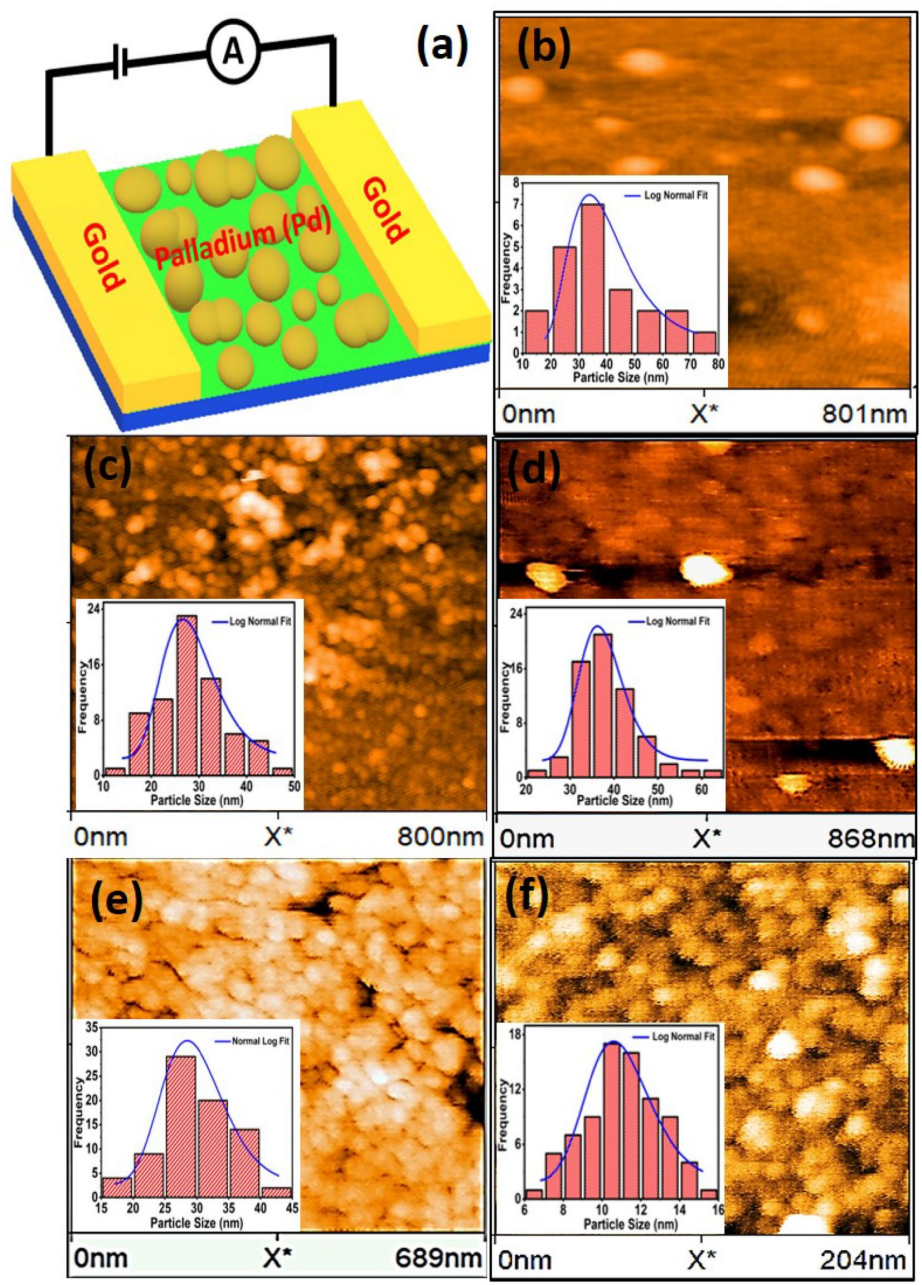
(**a**) represents a schematic diagram of contact geometry for measuring resistance in island-like ultra-thin Pd film. Blue colour depicts the glass substrate while green denotes a SAM deposited on top of glass. AFM topographs of ultra-thin films grown with (**b**) 3 nm, (**c**) 4 nm, (**d**) 4.5 nm, (**e**) 4.75 nm and (**f**) 5 nm thickness. Inset of each shows a log-normal distribution of grains obtained using the Image J software.

Images displayed in Fig. 2a,c,e respectively. The SAED images are marked by the presence of concentric ring-like structures denoting the formation of polycrystalline nano-island like ultra-thin films denoting short-range ordering. Four rings have been marked as representing (111), (200), (220) and (311) planes confirming the fcc crystal structure of metallic Pd (JCPDS file no. 87-0638).

To gain more insight into the morphology of the grown thin films, we made AFM measurements on each film. Figure 3b–f show representative AFM images of the nanoisland like ultra-thin films that were made with deposition times of (b) 3 min. (c) 4 min. (d) 4 min. 30 s. (e) 4 min. 45 s and (f) 5 min. Well defined grains of nanometer size, similar to^13^ can be clearly seen in all the images. From the images, an average grain size can be deduced as shown in the inset of all the Fig. 3b–f which shows a histogram of the frequency variation with the particle size. Blue continuous curves in each figure is a log-normal fit to the frequency data from which an average particle size for each deposition time was calculated. The grain size in each Fig. 3b–f was calculated by using an image processing software, Image J.

**Figure 4:**
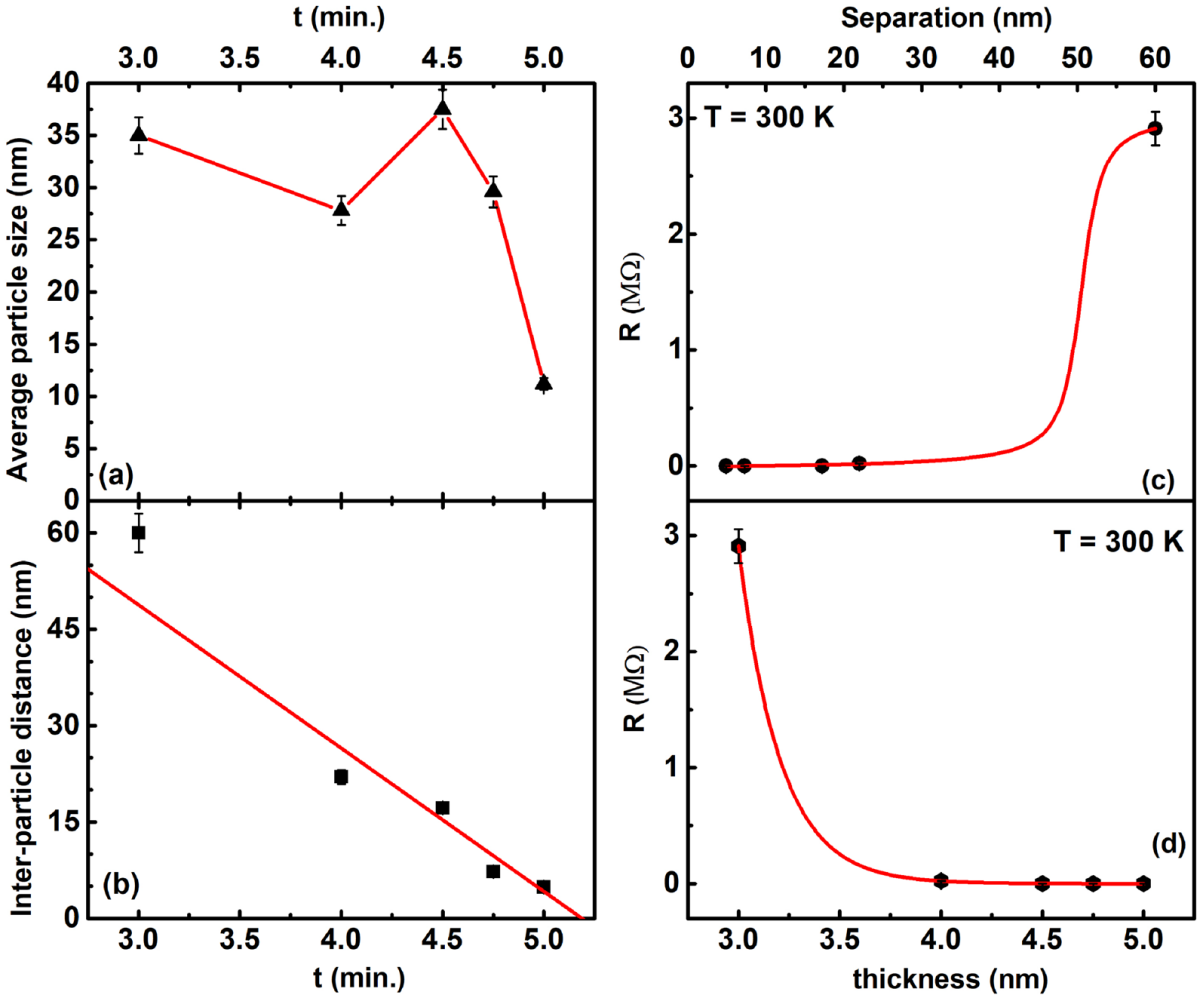
(**a**) Variation of average particle size with time (in minutes). Filled black triangles represent the average particle-size obtained from the log-normal fits to the AFM data of Fig. 3. Red solid curve is a guide to the eye. (**b**) Filled black squares correspond to the inter-particle separation obtained from the AFM images of Fig. (3). Red solid curve is a straight line fit to the data. From the fit, the slope m was obtained as 22.3 and the intercept c as 115.7. (**c**) Filled circles represent room temperature resistance variation with inter-island separation of films made by varying the deposition time. Red solid curve is a fit to the Langevin’s function (see text for details). (**d**) Filled hexagons correspond to thickness variation of resistance measured at room temperature in ultra-thin films of Pd. Red solid curve is a fit to Eq. (2).

Figure 4a shows the variation of the average particle size, as calculated from AFM images above, with the deposition time used in growing the nano-island ultra-thin films. It can be observed that the average particle size has a non-monotonic variation with the deposition time used, wherein, the average particle size first increases from $\sim$ 11 nm for a deposition time, *t*, of 5 min to $\sim$ 37.5 nm at *t* = 4.5 min With a further decrease in the deposition time, the average size decreases to 27.8 nm at *t* = 4 min before increasing again to 35 nm at *t* = 3 min. The grown thin films were made using a sputtering technique employing very low powers of the order of 5 W, such that the sputtered material has low energies and can get deposited on the substrate by condensation of islands of agglomerated Pd atoms. Such a non-monotonic behaviour of the average size, *r*, with the deposition time and, consequently the thickness, is reminiscent of the behaviour of condensation of water-vapour on a substrate where a similar non-monotonic behaviour with size has been observed^40^. It was found that if the substrate temperature is not very high (300 K) then a Brownian motion is inhibited and the metal aggregates behave like liquid droplets. The size, number density, morphology etc. of the metal aggregates depends critically on various processing conditions of the thin film growth. It is known that the metal droplets condense and grow on substrates by a combination and interplay of various processes and energy scales^40–42^. The processes involve surface adsorption of atoms forming adatoms, their interaction with each other and with previously existing islands, their binding to the surface governed by the binding energy etc. The ratio of the bulk cohesive energy, E$_c$, of the vapourised material to the adsorption energy on the substrate, E$_A$, determines the formation of either highly coalesced clusters (for E$_c$ > $E_A$) **or thin films form (for E**$_A$ > $E_c$)^40–43^. The slow transport of adatoms to random cluster sites from randomly nucleating sites result in a kinetic hinderance to droplet formation. The surface diffusion rate, *D*, was then found to vary as $D \sim$ exp((E$_W$-E$_A$)/k$_B$T) where E$_W$ corresponds to the energy of the most weakly bound surface site and k$_B$ is the Boltzmann’s constant^43^.

Therefore, at low deposition times, the metal droplets condense and grow without any interaction between the droplets. As the deposition time increases such that the surface coverage increases, the interaction between the droplets increases coalescing them and decreasing the size. Therefore, the initial decrease in the average particle size, *r*, from 35 nm at 3 min deposition time to 27.8 nm at *t* = 4 min may be a representation of the coalescence between the Pd islands such that E$_c$ wins over E$_A$. However, the subsequent increase in the average particle size of the Pd islands to 37.5 nm indicate that as the deposition time increases further, newer larger sized islands grow at different parts of the substrates. The subsequent sharp decrease of the Pd island size from 37.5 nm at 4.5 min to 11 nm at 5 min is indicative of the fact that the inter-island distance between Pd islands reached a critical value where small islands started to appear between the relatively smaller number of large sized Pd islands^40–42^. With an increase in deposition time further, the inter-particle separation should reduce to zero and a thick film would result with E$_A$ winning over E$_c$.

It would be instructive to know how the inter-island separation vary with the deposition time in the films grown above. The average inter-island separation for 3, 4, 4.5, 4.75 and 5 min of deposition was found to be 60 nm, 22 nm, 17.2 nm, 7.3 nm and 4.9 nm respectively. Figure 4b plots the variation of the inter-particle size, estimated from the AFM images of Figs. 3b–e, with time. It can be seen that the inter-particle separation decreases monotonically with time. In fact, a straight line denoted by red solid curve in Fig. 4b, was found to fit the data rather well.

In order to understand the charge transport mechanism in the grown ultra-thin films, it is prudent to check the room temperature variation of the resistance with the inter-particle separation in the nano-island films grown above, as shown by the filled black circles in Fig. 4c. It can be seen that the resistance decreases substantially as the inter-particle separation decreases. The film with inter-particle separation of 60 nm was very highly resistive at 3 M$\Omega$. As the inter-particle separation decreased to 22 nm, the resistance dropped to 22.5 k$\Omega$. The film with inter-particle separation as 4.9 nm was found to have a resistance of 3 $\Omega$, a six order of magnitude drop from the value for the 60 nm inter-particle separation film! This observation, then, suggests that a huge change in the charge transport of Pd ultra-thin films could be obtained just by varying the inter-particle separation obtained by varying the deposition time.

Red curve in Fig. 4c is a fit to the Langevin’s equation:

1 $$\begin{aligned} R(x)=R_0 + c \times \bigg (coth(x-x_c)-\frac{1}{x-x_c}\bigg ), \end{aligned}$$

where *R* is the resistance of the films, *x* is the average inter-particle separation, R$_0$ is a resistance offset, c is a constant and x$_c$ is the value of the inter-particle separation at which the resistance reaches its half value (1.5 M$\Omega$) when compared to the saturated value (3 M$\Omega$). From the fit, x$_c$ is 50.5 nm.

From Fig. 4c, it can be seen that the Langevin’s function fits the data rather well. Such a function is known to describe the magnetisation of paramagnets very well when the magnetisation of randomly oriented spins of a paramagnet increases on application of an external magnetic field, aligning the spins in the direction of the field and increasing the magnetisation consequently^44^. At the saturation value of the field, all moments are aligned and maximal magnetisation achieved. Similarly, Langevin’s function is also known to describe the polymerisation of polymers where the monomeric units of the polymeric chain increase in their length *L* on application of an external force until all the monomers are aligned in the direction of the force and maximum polymerisation achieved^45,46^. It was found recently^47^ that Langevin’s function described the resistance variation of inter-cluster separation of nanocluster assembled films very well. A good fit to the resistance variation of the inter-particle separation in our ultra-thin films, then, suggests that the thin films could be comprising of similar nanoclusters of Pd as that obtained in Ref.^47^. Xiong et al. calculated the variation of the electron density between two silica films as the distance between the films increased and found the electron density to vanish at distances lower than 4 $\AA$^48^. The expected small distance loss of wave-function overlap for insulating silica suggests that the used glass substrate may not be involved in the charge transport mechanism of the grown ultra-thin films. For a metallic film, the region of wave-function overlap is expected to be larger. So, the observed increase in the resistance of the films in Fig. 4 with an increase in the inter-particle separation may be due to a decrease in wave-function overlap of the charge carriers between the nano-islands such that maximal resistance is achieved in films where the nano-islands are so far apart that the charge carriers are localised at a given island resulting in a highly insulating film.

It is expected that in the thin film form, surface scattering contributes quite a bit to the resistivity of the sample, apart from the grain-boundary scattering^8,13,15–18,49^. To see if this is the case with our ultra-thin films, we plotted the thickness variation of resistance of these thin films, as shown by the filled black hexagons in Fig. 4d. Since surface scattering is known to vary as exp(-$\kappa$Hd), where $\kappa$ = h/$\lambda$; $\lambda$ is the mean free path and *H* is a function of thickness *d*, we fitted the experimental data to the expression:

2 $$\begin{aligned} R(d) = A \times \bigg (\frac{1}{1-exp(- B \times d)} \bigg ), \end{aligned}$$

where *A* is a constant and *B* is a product of $\kappa$ and H. From Fig. 4d, it can be seen that a reasonable fit has been obtained to the thickness dependence of resistance, implying that surface scattering is an important scattering mechanism for our ultra-thin films, apart from the grain boundary scattering.

### Charge transport variation in different films

**Figure 5:**
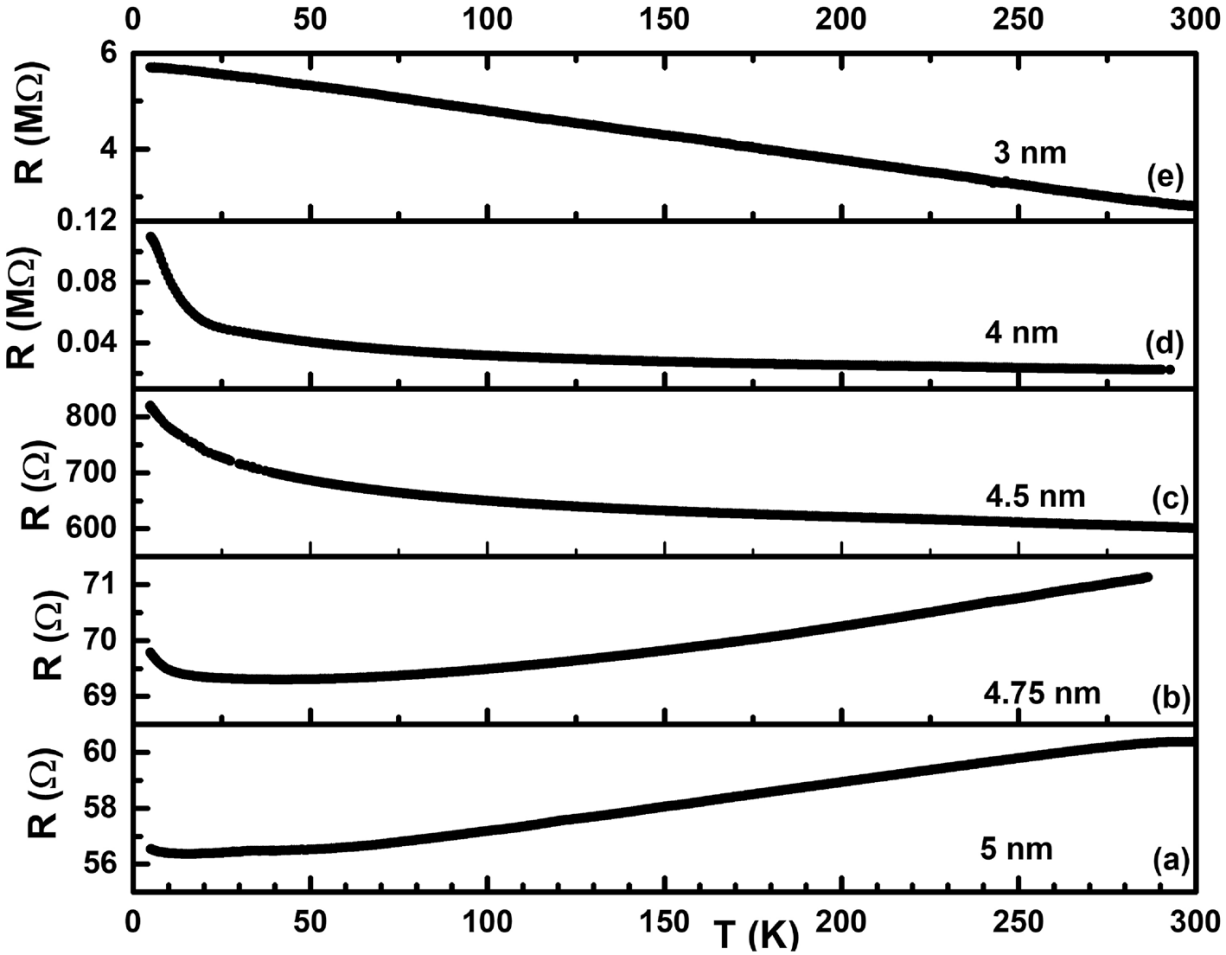
Temperature variation of resistance in films of thickness (**a**) 5 nm, (**b**) 4.75 nm, (**c**) 4.5 nm, (**d**) 4 nm and (**e**) 3 nm.

Since the room temperature resistance of our ultra-thin films vary appreciably with inter-cluster separation, the nature of charge transport in each of these films is expected to be widely different from each other. To investigate the details of the charge transport mechanism in the films, we did a temperature variation of resistance in the ultra-thin films as shown in Fig. 5a–e. From the figures it is quite clear that the temperature variation of resistance, and consequently charge transport, is very different in the films. The films of thicknesses 5 nm and 4.75 nm were found to display a positive slope (dR/dT > 0) in the R–T curve over a large temperature range of 300–50 K, implying a metallic charge transport in this temperature range in this film. In the 5 nm thin film, the resistance almost saturates to a residual resistance for temperatures below 50 K but shows a small upturn below 10 K. In contrast, in the 4.75 nm thin film, the slope of the R-T curve changes to negative (dR/dT < 0) below 50 K, implying an insulator behaviour below 50 K. This behaviour is reminiscent of similar behaviour of charge transport observed in thin films of Pd^13^ where a metal to insulator transition happened as the temperature of the system changed. In contrast, the films of thicknesses 4.5 nm, 4 nm and 3 nm were resistive (dR/dT < 0) in all the measured range of temperatures.

The seminal model that describes the temperature dependence of electrical resistance of a three dimensional monovalent metal with a spherical Fermi surface is that of Bloch–Grüneisen^28^ which ascribes it to arising from scattering of electrons due to acoustic phonons. The contribution to resistance from such a model is given by:

3 $$\begin{aligned} \rho = \rho _0 + \rho _{BG}(T), \end{aligned}$$

where

4 $$\begin{aligned} \rho _{BG}(T) = A\frac{T^n}{\Theta ^n}\int _{0}^{\frac{\Theta }{T}}\frac{x^n dx}{(e^x - 1)(1-e^{-x})}. \end{aligned}$$

Here, $\rho _0$ is the residual resistance that is temperature independent and is dependent on scattering arising from electrons scattering off defects, impurities and disordered regions^5,49,50^. From the discussions of Fig. 4d above, surface scattering plays a dominant role in the residual resistivity of our ultra-thin films. *A* is a constant that is proportional to $\lambda _c \omega _D$/$\omega _p^2$ where $\lambda$, $\omega _D$ and $\omega _p$ are electron–phonon coupling constant, Debye frequency and plasma frequency respectively. $\Theta$ in Eq. (4) is a characteristic temperature that is expected to match with the Debye temperature $\Theta _D$ obtained from specific heat measurements^51–53^.

Equation (4) is a generalised Bloch–Grüneisen expression where the exponent *n* depends on the dominant scattering mechanism at play^49,52,54,55^. While a *n* = 2 value is ascribed to a dominant electron–electron scattering mechanism^54,56^, a *n* = 3 value to s-d electron scattering (Bloch–Wilson mechanism)^55^, a *n* = 4.5 value to electron–magnon scattering^54^ and a *n* = 5 value to electron-acoustic phonon scattering^5,49,52,54,55^. In a three-dimensional bulk metallic palladium, the temperature variation of resistance is known to be of T$^5$ kind implying a dominant electron-acoustic phonon scattering mechanism. However, it is possible that other scattering mechanism maybe at play once the dimension of the metal is reduced. To investigate this, we added another scattering term to the resistance expression (4), similar to that used by Matula et al.^51^ and Joseph et al.^53^ of the form below:

5 $$\begin{aligned} \rho _{BG}(T) = \rho _0 + A\bigg [1+B\bigg (\frac{\Theta }{T}\bigg )^p\bigg ]\bigg (\frac{T}{\Theta }\bigg )^5\int _{0}^{\frac{\Theta }{T}}\frac{x^5 dx}{(e^x-1)(1-e^{-x})}, \end{aligned}$$

where *B* is another constant and *p* denotes the difference from the value 5 indicating the additional scattering mechanism that maybe at play apart from the electron-acoustic phonon kind of scattering mechanism.

**Figure 6:**
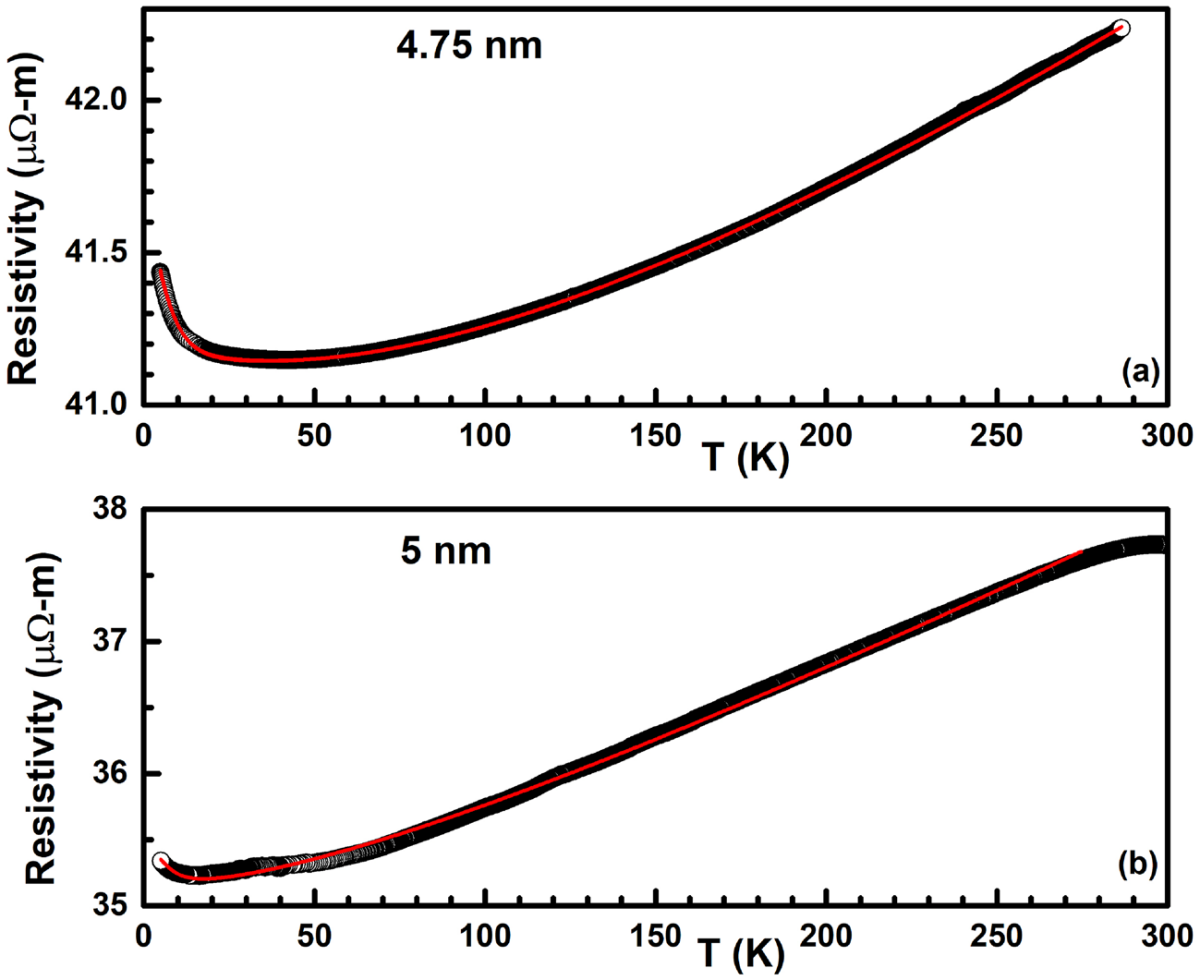
Temperature variation of resistivity of the (**a**) 5 nm and (**b**) 4.75 nm thin film. Black open circles denote the experimentally obtained data points while the red curve is a fit to Eq. (6) (see text for details).

Figure 6**a** shows the evolution of resistivity in the 4.75 nm thin film as the temperature is decreased from 300 K to lower temperatures. The resistivity is seen to steadily decrease with a decrease in temperature (dR/dT > 0) till $\sim$ 50 K, implying a metallic charge transport in this temperature interval. However, instead of reaching a residual resistivity at the lowest measured temperature, one can observe an upturn in resistivity below $\sim$ 50 K (dR/dT < 0), signalling a metal-insulator transition in the 4.75 nm thin film at $\sim$ 50 K. In order to explain similar observations in thin metallic films, Al’tshuler et al.^2,3^ investigated the influence of interference between conduction electrons and elastic scattering of these electrons with defects in the sample and found that the interference leads to an additional term in the resistance that has a square root temperature dependence. In order to model this, the expression (5) was modified to:

6 $$\begin{aligned} \rho = \rho _{BG} - \gamma _{ee}\sqrt{T}, \end{aligned}$$

where $\gamma _{ee}$ is the Al’tshuler’s parameter and is a constant. Red solid curve in Fig. 6a is a fit to Eq. (5). It can be seen that the fit to the experimentally obtained data is excellent. From the fit, $\gamma _{ee}$ was obtained as 0.47709 while the electron-acoustic phonon parameter *A* was obtained as 3.4 eV/$\AA ^2$, very close to the bulk value of 3.665 eV/$\AA ^2$ for fcc Pd^57^. Similarly, the Debye temperature $\Theta$ was also found to be 70.8 K, much lower than the bulk value of 275 K^51^. The value of the exponent *p* was 0.55, implying an additional scattering mechanism that has a T$^{4.45}$ kind of temperature dependence in this thin film. Since the exponent 4.45 is very nearly equal to 4.5, it suggests an additional electron–magnon scattering in this Pd thin film. Bulk palladium is known to be non-magnetic but is close to fulfilling the Stoner’s criterion of ferromgnetism (N(E$_F I$) > 1)^58^ where N(E$_F$) is the density of states at the Fermi level E$_F$ and *I* is the Stoner’s parameter having a value of 0.87 for Pd^57^. However, in the nanoscale, palladium has been shown to exhibit ferromagnetism^58–60^. Since our ultra-thin films have grains that are nanometer sized (see Fig. 3), it is quite probable that such nanometer sized films are ferromagnetic in nature contributing to an additional electron-magnon scattering in the temperature dependence of resistance.

Figure 6b plots the temperature variation of resistivity in the 5 nm thin film. It can be observed that the temperature variation in this film has a similar behaviour as that of 4.75 nm film, namely, decrease of resistance with decrease in temperature (dR/dT > 0) over a large temperature interval. Similar to the 4.75 nm thin film, we can observe a metal-insulator transition in this film as well but at a lower temperature of 20 K. However, the data quality is not as good as that in the 4.75 nm and we observe a slight hump   30 K which is an experimental artefact. Red solid curve in Fig. 6b is a fit to Eq. (6) that gave $\rho _0$ = 35.34 $\upmu \Omega$-m, *A* = 3.4 eV/$\AA ^2$, *B* = 0, $\Theta$ = 51.18 K and $\gamma _{ee}$ = 0.15. Both the Debye temperature $\Theta$ as well as the Al’tshuler’s parameter $\gamma _{ee}$ are found to decrease in the 5 nm thin film when compared to the 4.75 nm thin film.

**Figure 7:**
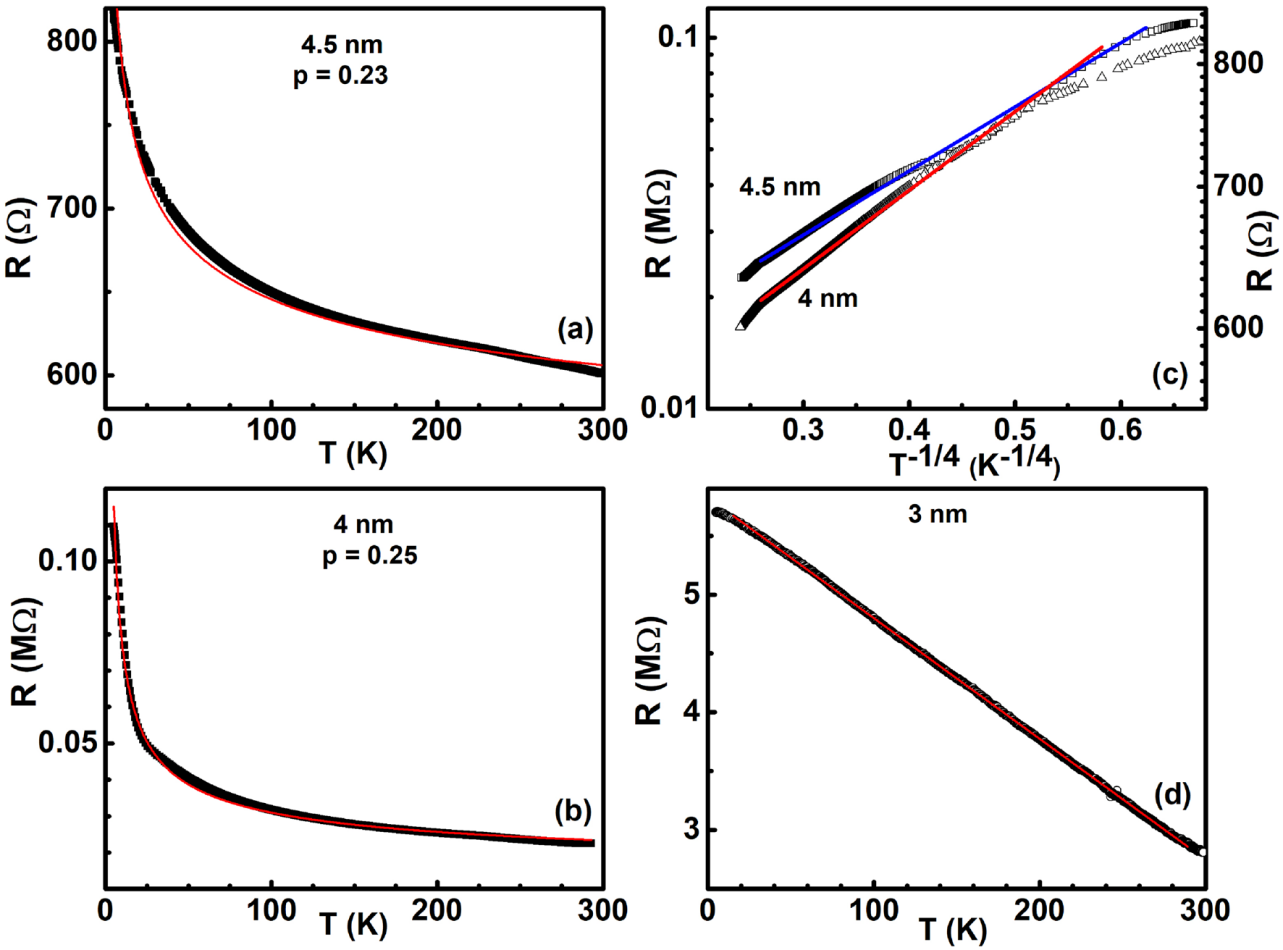
Black filled squares denote the temperature variation of resistance in (**a**) 4.5 nm and (**b**) 4 nm thin films. Red solid curves in each is a fit to Mott’s variable range hopping (See text for details). (**c**) log-linear variation of resistance with T$^{-1/4}$ in films of thickness 4.5 nm represented by open triangles and 4 nm represented by open squares. Blue and red curves are straight line fits to the data corresponding to 4.5 nm and 4 nm thin film respectively. (**d**) Open circles represent the temperature variation of resistance in the 3 nm ultra-thin film while the red solid curve is a straight line fit to the data.

Unlike the 5 nm and 4.75 nm thin films that exhibited a metallic charge transport in a large temperature interval, the 4.5 nm and 4 nm thin films were found to be insulating in the whole temperature range as can be seen from the negative slope of the temperature dependence of resistance of such films (dR/dT < 0) in Fig. 7a,b. Red solid curves in Fig. 7a,b are fits to Mott’s variable range hopping model of insulating charge transport that is understood to arise due to thermally activated hopping of electrons transiently localised at a quantum level to another level around the Fermi level E$_F$^61,62^. The expression for Mott’s variable range hopping resistance is given by:

7 $$\begin{aligned} R = R_0(T_0/T)^q, \end{aligned}$$

where *q* is the hopping exponent and takes the value 1/3 and 1/4 in a two-dimensional and three-dimensional system respectively.

From the fits to the resistance data in Fig. 7a,b, the exponent was found to have the values 0.23 and 0.25, respectively. This observation, then, implies that as the thickness of the film is reduced to 4.5 nm and 4 nm, an insulating charge transport happens in palladium thin films that is of a three dimensional Mott’s variable variable range hopping kind. At the thickness of 4.5 nm and 4 nm, the average inter-island separation of the films was found to be 17 nm and 22 nm respectively (see Fig. 4). It is expected that as the distance between the islands increases, the metallic islands of Pd get separated from each other by Coulomb-like energy barriers^63^ limiting the conductivity of the initially metallic system. At large inter-island separation of 17 nm and 22 nm, the energy barriers seem large enough to reduce the conductivity of the films and result in weak localisation of the electrons on the metallic islands^3^. At such inter-island separation, dephasing of the electron wave-function may happen due to inelastic scattering of the electron with various defects like surfaces, grain boundary etc. present in the ultra-thin film. The Mott kind of charge transport, then, implies that the transport happens not only by transition between single quantum levels of each metallic island but primarily between group of levels that form metallic islands and are separated by Coulomb-like barriers.

To confirm that the obtained fitting exponent is 1/4 for the 4.5 nm and 4 nm films over the entire temperature range, we replotted the resistance data of Fig. 6a,b as ln R vs. T$^{-1/4}$, shown as open triangles and squares respectively in Fig. 7c. Red and blue solid lines are straight line fits to the data corresponding to 4 nm and 4.5 nm films respectively. It can be seen that the straight line fits the resistance data of the 4 nm film quite well in a large temperature range. This is expected since the fitting exponent *q* from Fig. 7b was obtained as 0.25 in this film. On the other hand, it was found that a straight line fits both the high temperature data as well as the low temperature data of the 4.5 nm film quite well. However, there was a deviation to the straight line fit in the intermediate temperature range. One possible reason could be the fact that the resistance change in the 4.5 nm film from room temperature to 4 K is not by orders of magnitude (they are not very highly insulating), unlike the 4 nm thin film which has a sharper change of slope at low temperatures (see Fig. 7b). So, it is possible that a ln R variation with T$^{-1/4}$ is not linear in such films.

In contrast, the resistance in the 3 nm film was found to decrease linearly with a decrease in temperature in the entire measured range of temperature! Black open circles in Fig. 7d represent data points while the red continuous curve is a straight line fit to the data. It can be seen that the straight line fits the data very well. To our knowledge, such a linear decrease of resistance in an insulating resistive response is observed for the first time.

To check the self-consistency of the obtained exponents for the 4.5 nm and 4 nm films and to find if the Efros–Shklovskii^64,65^ mechanism of charge transport may have any bearing on the insulating transport observed above, we performed a resistance curve derivative analysis (RCDA) according to which, one calculates the logarithmic derivative of resistance as^47,66–68^:

8 $$\begin{aligned} W = -\frac{\partial ln R(T)}{\partial ln (T)} = p\bigg (\frac{T_0}{T}\bigg )^p. \end{aligned}$$

A plot of *lnW* vs. *lnT*, then, gives the hopping exponent *p* since *lnW* = $A - p*lnT$. For an Efros-Shklovskii charge transport, *p* = 1/2.

**Figure 8:**
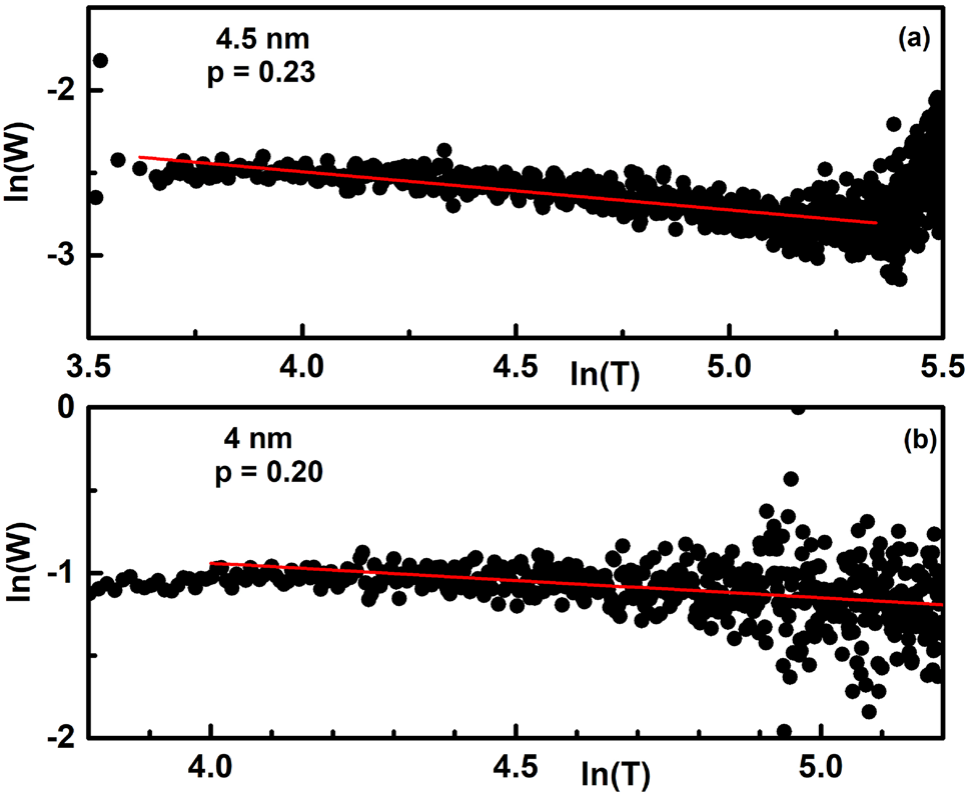
Filled circles denote the *lnW* variation of *lnT* in (**a**) 4.5 nm and (**b**) 4 nm thin film. Red solid lines in each panel is a straight fit to the respective data.

Figure 8a,b depict the variation of *lnW* with *lnT* in films of thickness 4.5 nm and 4 nm respectively. In order to estimate the hopping parameter *p* in each case, a least square straight line fit was made to each data, as shown by the red thick line. From the fits, *p* was obtained as 0.23 and 0.20 for the 4.5 nm and 4 nm thin films respectively. It can be observed that the value of the hopping exponent matches exactly with the one obtained from Fig. 7 for the 4.5 nm but is slightly different for the 4 nm film. However, the obtained values are in close proximity to the value 1/4 expected for a Mott kind of charge transport in *D* = 3 dimensions, confirming that the charge transport in our insulating films are of the Mott’s variable range hopping kind and not the Efros–Shklovskii kind, implying that the hopping energy is greater than the gap energy in these thin films^66^.

**Figure 9:**
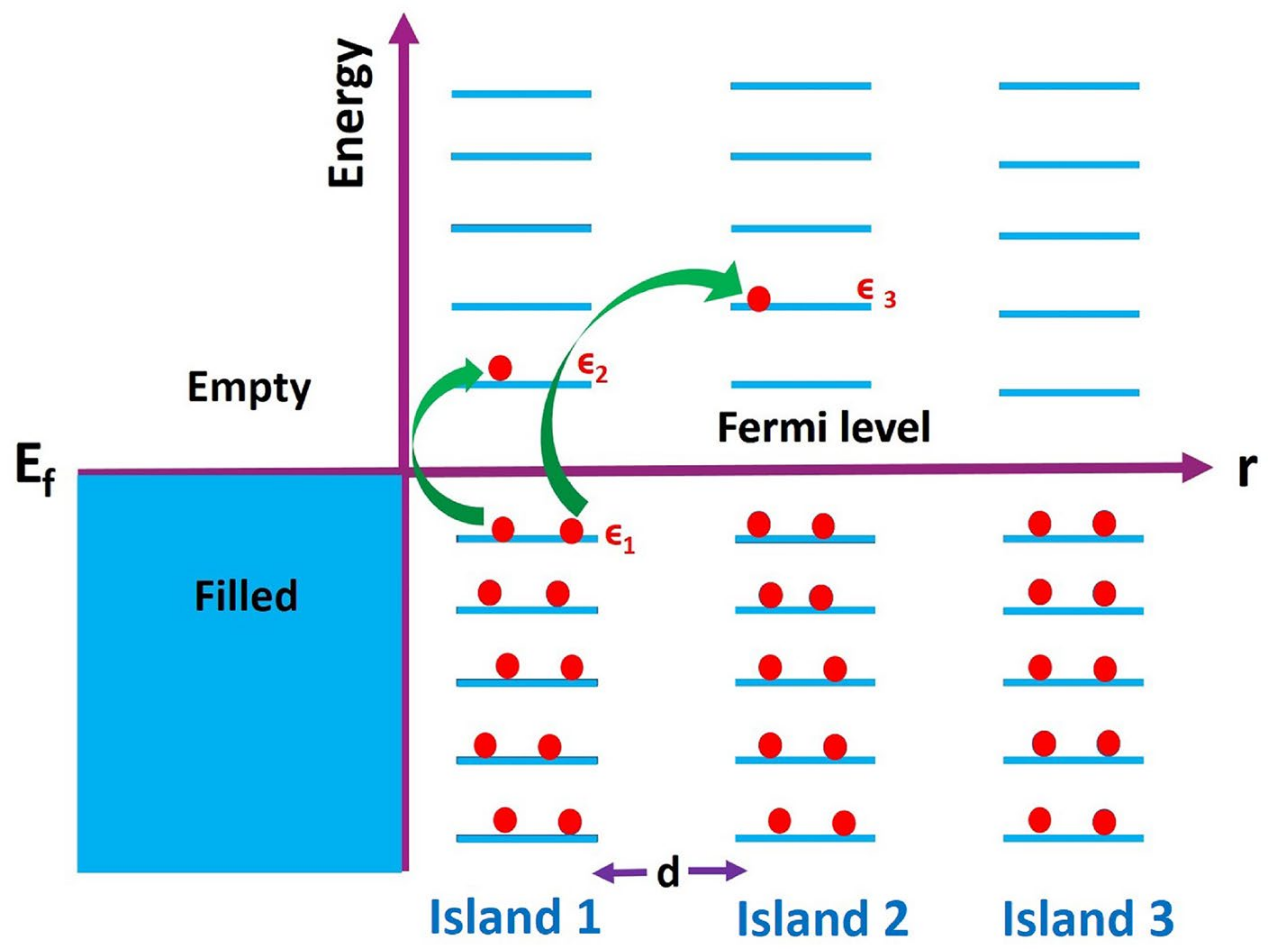
Schematic of Mott’s variable range hopping mechanism in nanoisland films of palladium that is a bulk metal. Two hopping events are shown by curved lines. The first hopping event is between two states $\epsilon _1$ and $\epsilon _2$ of the same island 1. The second hopping event is from $\epsilon _1$ and $\epsilon _3$ of islands 1 and 3 respectively.

In an effort to understand the details of the charge transport described above more clearly, we have made a schematic of the phenomenon in Fig. 9 which shows the transformation of the energy band diagram of the bulk palladium metal when palladium is made into a quasi-continuous nano-sized ultra-thin film. Left side of the schematic shows a partially filled band of a bulk palladium metal such that the Fermi level E$_F$ lies within the band and the energy levels within the band are of the order of 10$^{-23}$ eV, so that they form a continuous band^69^. As soon as the bulk palladium is transformed into a ultra-thin film that comprises nano-sized islands that are separated from each other by few nanometers, the quasi-continuous energy levels of the bulk band (shown in left) transform to discrete energy levels that are shown in the right side of the figure. The schematic shows energy band diagram in three representative islands out of the many islands, for clarity.

Charge transport in disordered semiconductors has been described by the Mott’s variable range hopping mechanism, wherein, charge transport of an electron transiently localised at a quantum state of energy $\epsilon _1$ in the valence band to another localised quantum state $\epsilon _2$ in the conduction band happens by absorption of a phonon of energy $\epsilon$ that equals the energy difference of $\epsilon _1$ and $\epsilon _2$^61,62^. Unlike a semiconductor, in a bulk metal like palladium, there exist no such localised states within bands. However, due to quantum size effects, the energy levels within a band show up their discrete character as shown in Fig. 9 above. Hence, charge transport in such nano-island films may happen by thermally assisted hopping of electrons that are localised on such levels transiently, similar to that in semiconductors. A hopping event between states $\epsilon _1$ and $\epsilon _2$ within the same island is shown by a curved arrow in Fig. 9. Since the probability of Mott’s variable range hopping is critically dependent on the ratio of the distance *d* between the states and the localisation length $\xi$, the charge transport in our palladium films would also be governed by the distance *d* between the islands. For shorter distances, charge transport may happen via transition between states $\epsilon _1$ of the first island and $\epsilon _3$ of the second island as shown by the second curved arrow in Fig. 9. However, the probability of such inter-island hopping decreases very fast as the distance between the islands increases.

### Charge transport under hydrogenation

From the above discussions, it is clear that various kinds of phenomenon like grain boundary scattering, surface scattering, electron acoustic-phonon scattering as well as electron–magnon scattering affect the charge transport of the ultra-thin Pd films that we have grown. It would be instructive to know if the charge transport described above could be manipulated further by some other external parameter that can be easily tuned and controlled. Hence, we decided to study the room temperature differences in the charge transport behaviour of the ultra-thin films of Pd by subjecting them to low concentrations of hydrogen gas since it is very well known that Pd’s surface acts catalytically on incoming H$_2$ gas to convert it into H atoms that diffuse into the octahedral lattice of Pd to form a palladium hydride PdH$_x$^19–25^.

**Figure 10:**
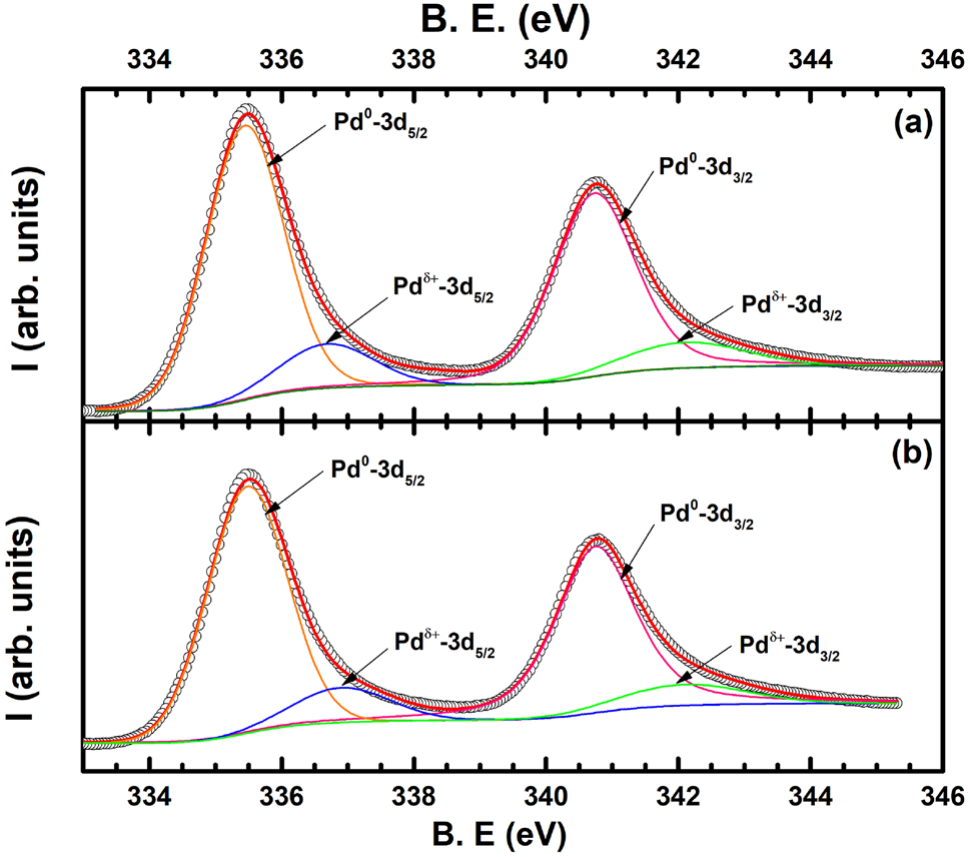
XPS spectra of the 5 nm thin film (**a**) before and (**b**) after exposure to 5000 ppm of H$_2$ gas for 1 hour. Open black circles denote the data while the thick red solid line is a fit to the data. Thin solid lines in each denote the spectral decomposition of the spectra in peaks corresponding to Pd$^0$-3d$_{5/2}$ (orange), Pd$^0$-3d$_{3/2}$ (pink), Pd$^{\delta +}$-3d$_{5/2}$ (blue) and Pd$^{\delta +}$-3d$_{3/2}$ (green).

X-ray photoelectron spectroscopy (XPS) is a powerful technique to probe the changes in the elemental constitution of a material by measuring the changes in the binding energy of a given element according to the changes in the local environment of the given element^36,71–73^. Therefore, to confirm the formation of PdH$_x$ in the ultra-thin films of Pd at low concentrations of hydrogen, we subjected the thickest 5 nm film to a low concentration of 5000 ppm of H$_2$ gas and measured the changes in the associated XPS spectra of the thin film. Figure 10a shows the XPS spectra of the as-grown Pd thin film where the open black circles denote the data while the superimposed red thick curve is a fit to the experimental data using the XPS peak software. The experimental data was resolved to show the presence of two main peaks characteristic of 3d levels governed by spin-orbit coupling. The first resolved peak (shown in orange) at the lower binding energy (B.E.) of 335.4 eV is ascribed to the 3d-$_{5/2}$ spin-orbit level of Palladium in the metallic state (Pd$^0$)^70,71^ while the second resolved peak (shown in pink) at the higher B.E. of 340.8 eV corresponds to the Pd$^0$-3d$_{3/2}$ spin-orbit level^36^. The intensity ratios of the deconvoluted peaks occur roughly in the expected ratio of 3:2, corresponding to the multiplicity of the spin-orbit split features. It can also be observed from Fig. 10a that two additional small peaks occur at B.E’s of 336.6 eV and 342 eV. It was found that chemisorption of oxygen (O) atoms on the surface of Pd nanoparticles result in a shift of   1.2 eV from the Pd$^0$ peak^72,73^. Since we applied a layer of self assembled monolayer (SAM) on the glass surface in order to reduce the stiction of Pd on glass, the two small peaks could be arising due to Pd’s binding to the O atoms of the SAM.

Figure 10**b** shows the changes to the XPS spectra of the 5 nm thin film after it was hydrogen loaded for 1 h. The colour coding of the spectral decomposition is the same as that obtained in Fig. 10a. It was found that the peaks corresponding to both the Pd$^0$-3d$_{5/2}$ spin-orbit level shifted by + 0.1 eV, in agreement to those obtained in thin films of Pd exposed to hydrogen^74^. However, the shift in the B.E.’s of the two smaller peaks was found to be higher at + 0.3 eV.

**Figure 11:**
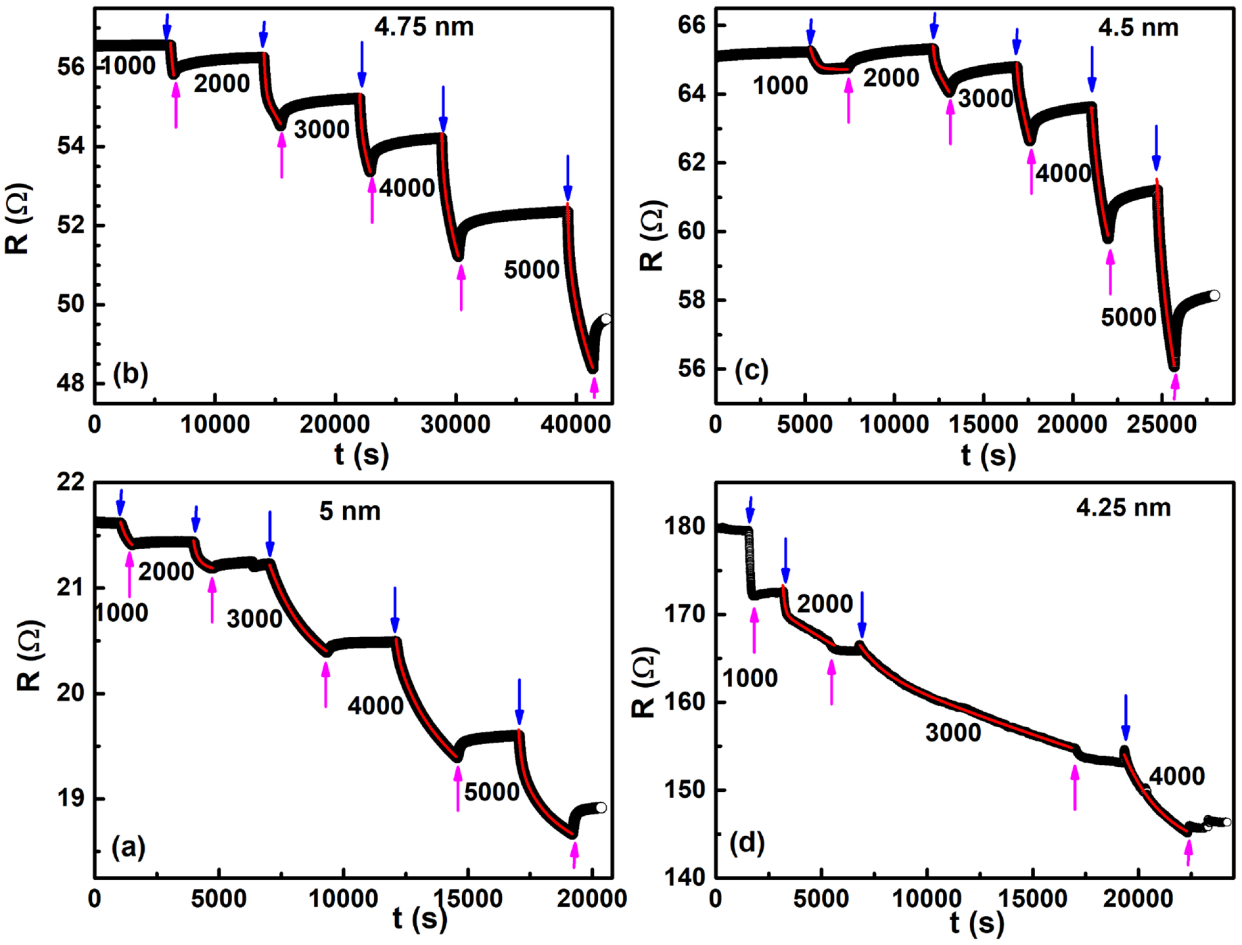
Change of resistance after subjecting low concentration hydrogen in ultra-thin films of Pd at 298 K with thickness varying as (**a**) 5 nm, (**b**) 4.75 nm, (**c**) 4.5 nm and (**d**) 4.25 nm. Blue colour arrow denotes the loading of hydrogen gas while the pink arrow shows the unloading of hydrogen gas at a given instance of time. Values of low concentration hydrogen gas varying from 1000 to 5000 ppm are also shown.

Figure 11 shows the variation of resistance in ultra-thin films of Pd after they were subjected to low concentrations of hydrogen (H$_2$) gas at room temperature (298 K) and atmospheric pressure (1 atm) conditions. From Fig. 11a–c, it is clear that the resistance of a given ultra-thin film decreases after being subjected to low concentration H$_2$ gas. Barr et al. showed that when ultra-thin island like films of Pd with inter-island separation near the percolation threshold are subjected to low concentration H$_2$ gas, charge transport happens via the tunnelling mechanism^19^. There are two effects on the tunnelling mechanism on H$_2$ exposure: (i) for those hydrogen atoms that are adsorbed at the surface, the resistance increases due to an increase in the work function of the material and (ii) for the H atoms that are pushed inside from the surface into the Pd matrix, a lattice expansion of the fcc Pd lattice happens, the inter-island gap decreases resulting in a decrease of the initial resistance. Such a hydrogen absorption induced lattice expansion (HAILE) has been observed in meso wires of Pd^75^, nanofibers^76^, nanoclusters^77^ as well as in ultra-thin films of Pd^27,36^. Hence, the immediate decrease of resistance in ultrathin films of Pd on loading of H$_2$ gas of varying concentrations, as shown in Fig. 11a–c is ascribed to the HAILE phenomenon.

In contrast, in the thin film of 4.25 nm thickness (Fig. 11d), there is an initial increase in the resistance on hydrogen loading before the resistance starting to decrease. The time interval for the decrease of resistance is found to initially increase as the concentration of the hydrogen gas increases and then decrease afterwards. For instance, at 1000 ppm H$_2$, the increase of resistance happened till $\sim$ 11 s after hydrogen exposure and then started to decrease; for 2000 ppm R increased till 17 s before starting to decrease while for 3000 ppm, it increased till 73 s before the decrease. However, on further increase of H$_2$ gas to 4000 ppm, R increased till only 54 s after hydrogen loading before eventually starting to decrease.

Bulk Pd metal’s surface is very well known for its excellent catalytic response to an incoming H$_2$ gas molecule, wherein, they get broken to form two H atoms on the Pd’s surface according to the following reaction^36,78^:

9 $$\begin{aligned} H_2 + 2S_{Pd} \rightarrow 2H-S_{Pd}, \end{aligned}$$

where S$_{Pd}$ denotes the number of available surface Pd sites. The dissociated H atoms are then pushed inside the Pd matrix to form PdH$_x$. For x < 0.01, a solid solution of PdH$_{\alpha }$ is formed due to random occupation of the H atoms inside the Pd matrix while for 0.02 < x < 0.6, an octahedral lattice of PdH$_{\beta }$ gets formed^19–25^. Hence, in the very low concentration of 1000–5000 ppm of H$_2$ used on our ultra-thin films, a solid solution of PdH$_{\alpha }$ gets stabilised. An initial increase of resistance in the island-like 4.25 nm thin film of Pd (see Fig. 11d), then, suggests an increase in the work function of the 4.25 nm thin film due to the surface adsorbed H atoms on the available Pd sites^13,19–21,78^. From the discussions above, we have already seen that surface scattering plays a prominent role in our ultra-thin films. The increase in work function of Pd thin film implies formation of hydrogen anions at the Pd surface by taking in the conduction electrons of the Pd lattice. The loss of conduction electrons results in a lowering of the Fermi surface of the Pd thin film and a resultant increase of the work function^79^. Therefore, the initial increase in the resistance of the 4.25 nm thin film is attributed to surface scattering of the electrons with the hydrogen anions formed at the Pd surface.

Singh et al. considered ultra-thin films of Pd as comprising a network of grains that are near a percolation threshold. On formation of PdH$_x$, the grains expand and connect with each other providing a new percolation path resulting in a decrease in the value of the initial resistance. With the passage of time, more and more new percolative paths open up lowering the resistance further. In order to model the decrease of resistance with time, it has been proposed that the resistance decrease on exposure to hydrogen gas happens via two time-constants $\tau _1$ and $\tau _2$^13^. The smaller time-constant $\tau _1$ indicates the opening up of the first new percolative path while the second time-constant $\tau _2$ is a longer one which denotes the slower opening up of the newer percolative paths. Hence, in order to understand the immediate decrease of resistance in ultra-thin films of 5 nm, 4.75 nm, 4.5 nm thickness and the subsequent decrease of resistance after an initial increase in resistance of 4.25 nm thin film, we fitted the resistance data of Fig. 11 to the equation:

10 $$\begin{aligned} R = R_a + R_b \times exp\bigg (-\frac{t}{\tau _1}\bigg ) + R_c \times exp\bigg (-\frac{t}{\tau _2}\bigg ) + R_d \times exp\bigg (-\frac{t}{\tau _3}\bigg ), \end{aligned}$$

where $R_a$ is the base-line starting resistance, $R_b$, $R_c$ and $R_d$ are constants and $\tau _1$, $\tau _2$ and $\tau _3$ are the time-constants that define the time taken by the final resistance to reach 1/e of its starting value. Red solid lines in Fig. 11a–d are the fits to equation 10. The obtained values of the time-constants $\tau _1$, $\tau _2$ and $\tau _3$ from the fits are tabulated in Table 1:

**Table 1:** Fitted values of time constants $\tau _1$, $\tau _2$ and $\tau _3$ obtained at different concentrations of H$_2$ gas for ultra-thin Pd films of varying thicknesses.

Thickness (nm)	Time-constant (s)	Concentration (ppm)				
		1000	2000	3000	4000	5000
5	$\tau _1$	373	75	–	90	103
	$\tau _2$		310	–	835	812
	$\tau _3$		–	1667	10,482	22,432
4.75	$\tau _1$	289	140	132	102	124
	$\tau _2$	–	–	3285	1582	1507
	$\tau _3$	–	–	–	–	11,926
4.5	$\tau _1$	276	105	97	–	253
	$\tau _2$	–	6396	3109	781	6759
4.25	$\tau _1$	–	91	1156	1996	
	$\tau _2$	–	351	67,429	–	
	$\tau _3$	–	14,852	–	–	

We found that in order to fit the resistance decrease with time, an additional time-constant $\tau _3$ is necessary. As described in the experimental section, we had applied a self-assembled monolayer (SAM) in an effort to reduce the stiction of Pd on glass and increase the switching action of Pd under the exposure to hydrogen gas. The presence of a third time-constant in the system indicates that some parts of the SAM may have been chipped off from the glass surface due to the sputtering process exposing the bare glass to Pd. Since Pd has a very high stiction on glass^80^, this would result in an additional large time-constant in the system, as observed.

A comparison of the time-constants reveal that the three time-constants differ by an order of magnitude from each other such that $\tau _1< \tau _2 < \tau _3$. It has been observed that the lattice expansion on exposure to hydrogen increases monotonically with the concentration from PdH$_0$ to PdH$_{max}$^81^. This suggests that lower concentrations of hydrogen would require longer times for the HAILE mechanism to set-in. From the Table 1, this has been found to be true statistically. The largest time-constant $\tau _3$ is not observed in the lowest concentration of 1000 ppm possibly because the time required to observe it is much larger than the time scale in which we measured the resistance decrease on hydrogen exposure. For the 4.25 nm thin film, even though the resistance decreased after the initial increase, the behaviour of the film was found to be quite different from the remaining films. The resistance drop at 1000 ppm was found to be almost linear in the measured time-scale. Hence, we did not fit it to the Eq. (10). Additionally, both for the 2000 ppm and 3000 ppm of hydrogen concentrations, the resistance kept on decreasing even after hydrogen unloading unlike the behaviour of other films where the resistance increased and tried to regain the starting value after hydrogen unloading (c.f. Fig. 11a–c).

**Figure 12:**
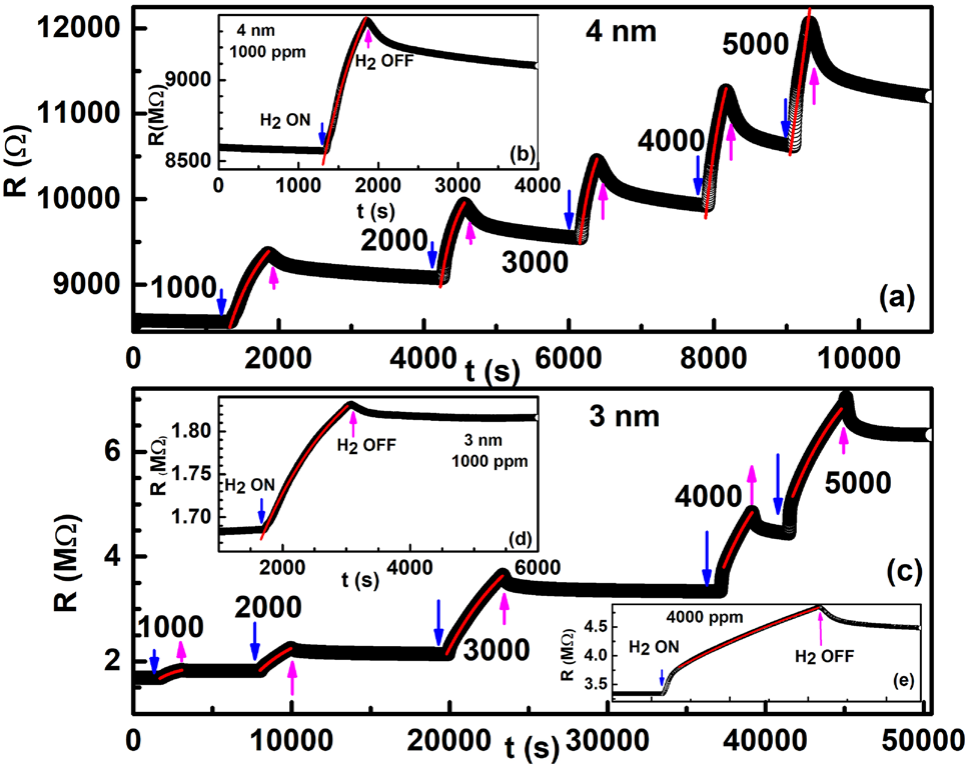
Main panel of (**a**) and (**c**) show the time dependence of resistivity in ultra-thin films of thickness (**a**) 4 nm and (**c**) 3 nm on exposure to hydrogen of varying concentrations. Insets (**b**) and (**d**) show an exponential fit (see text for details) on an expanded scale for clarity at a hydrogen loading of 1000 ppm. Inset (**e**) shows a straight line fit to the data at 4000 ppm H$_2$ concentration.

The behaviour of the time-dependence of resistance of the thinner films of thickness 4 nm and 3 nm was found to be completely different from those observed in Fig. 11 in the entire time range. Similar to the initial increase of the resistance with time observed in the thin film of thickness 4.25, the resistance was found to increase with time in the entire time range under exposures to low concentration hydrogen as shown in Fig. 12. Red curves in each figure is an exponential fit to the data as below:

11 $$\begin{aligned} R = R_0 + A(1-e^{-t/a}), \end{aligned}$$

where $R_0$ is the starting resistance value, *A* is a constant and *a* denotes the rise time constant. The values of the time-constants obtained from the fits are tabulated in the Table 2 below:

**Table 2:** Fitted values of time constant *a* obtained at different concentrations of H$_2$ gas for ultra-thin Pd films of thickness 4 nm and 3 nm.

Thickness (nm)	Time-constant (s)	Concentration (ppm)				
		1000	2000	3000	4000	5000
4	*a*	369	161	146	140	–
3	*a*	977	2194	3369	2560	2402

It can be seen that the time-constant shows a contrasting behaviour for the 4 nm thin film when compared to the 3 nm thin film. For the 4 nm thin film, the rise time-constant *a* decreases monotonically with an increase of concentration from 1000 ppm to 4000 ppm. When the film was exposed to a concentration of 5000 ppm, the rise was found to be linear rather than exponential in the measured time range. A straight line fit to the resistance data is shown in the inset (e) of Fig. 12. For the 3 nm thin film on the other hand, the time-constant *a* was found to display a non-monotonic behaviour with hydrogen concentration, wherein, *a* was found to increase on increasing hydrogen concentration from 1000 ppm until 3000 ppm above which concentration *a* decreased.

The increase in resistance on exposure to H$_2$ gas is ascribed to a work-function increase of the Pd thin films due to an increased adsorption of H atoms at the available Pd surface sites, as described above. The increase in time-constant *a* with decreasing H$_2$ concentration in the 4 nm thin film implies that the time taken for the surface adsorption of H atoms to result in an increase in work function, increases as the H$_2$ concentration decreases. From the discussions corresponding to Fig. 7, it was found that the charge transport in the 4 nm thin film was due to Mott’s variable range hopping mechanism. It is our expectation that the charge transport mechanism is unaltered on the exposure to H$_2$ gas of varying concentrations. Mott’s variable range hopping is dependent on the spatial separation of the islands^61,62^. From Fig. 4, it can be seen that the average spatial separation between the islands for the 4 nm thin film is $\sim$ 30 nm. At such large separation, it is reasonable to expect that the time taken for the Mott’s variable range hopping mechanism to set-up will increase as the concentration of H$_2$ decreases resulting in progressively lower number of H atoms to surface adsorb. However, the non-monotonic behaviour of the time-constant *a* for the lower thickness film of 3 nm is not understandable and it may have some correlation to the very unusual linear decrease of resistance with temperature (see Fig. 4d) observed in the 3 nm thin film.

## Conclusions

To conclude, we have performed a systematic investigation of the differences in charge transport mechanism of ultra-thin films of palladium that were made by sputtering technique. It was found that the thicker films were metallic in a large temperature range with a Bloch–Gruneisen mode of metallic transport. Below $\sim$ 50 K, these films were found to have a metal-insulator transition which was explained by Al’tshuler’s theory of increased resistance in disordered conductors. For the thinner films, the behaviour was insulating in the entire temperature range and Mott’s variable range mechanism of charge transport was found to hold true in such films. It was also found that hydrogen could be used as another independent control parameter for tuning the charge transport mechanism of the thin films. The room temperature resistance of the metallic films was found to decrease on hydrogenation of the films. It is proposed that such films may be at a percolation threshold and the resistance decrease ascribed to a hydrogen induced palladium lattice expansion. The increase in resistance of the insulating film, on the other hand, was found to be due to an increase in the work-function of such ultra-thin films. We believe that the systematic investigation of charge transport in thin films of palladium have wider implications for understanding differences in charge transport mechanism arising in metals when one dimension of bulk metals is reduced. Since palladium is proposed to be a very good hydrogen gas sensor that can be fabricated in a thin film form, the associated differences to the charge transport mechanism upon hydrogen gas loading and unloading can be used to design efficient hydrogen sensors with better time-constants. Our study also has implications for liquid metals whose properties vary by changing the amount of material that can be dissolved not only in the bulk but also on the surfaces^82,83^, similar to the hydrogen dissolution process in palladium. Film thickness dependent differences in the resistance change observed in our films above may help understand the differences of material dissolution process in liquid metals.
